# Improving Turnaround Time of Transabdominal Pelvic Ultrasounds with Ovarian Doppler in a Pediatric Emergency Department

**DOI:** 10.1097/pq9.0000000000000730

**Published:** 2024-05-27

**Authors:** Amanda S. Dupont, Patrick C. Drayna, Mark Nimmer, Shannon H. Baumer-Mouradian, Kendra Wirkus, Danny G. Thomas, Kevin Boyd, Sri S. Chinta

**Affiliations:** * From the Department of Pediatrics, Section of Pediatric Emergency Medicine, Medical College of Wisconsin, Milwaukee, Wisc.; † Department of Pediatrics, Section of Radiology, Children’s Wisconsin, Milwaukee Wisc.; ‡ Department of Pediatrics, Section of Radiology, Medical College of Wisconsin, Milwaukee, Wisc.

## Abstract

**Introduction:**

Adnexal torsion is an emergent surgical condition. Transabdominal pelvic ultrasound (US) with ovarian Doppler is used to diagnose adnexal torsion and requires a sufficient bladder volume. Reduce the turnaround time for US by 25% in girls 8–18 years of age who present to the emergency department (ED) for 24 months.

**Methods:**

Our baseline period was from January 2020 to June 2021, and the intervention period was from July 2021 to June 2023. Patients 8–18 years old who required an US in the ED were included. There are two key drivers: early identification of US readiness and expeditious bladder filling. Interventions were (1) bladder volume screening; (2) utilization of bladder volume nomogram to identify US readiness; (3) epic order panels; and (4) rapid intravenous fluid method. The primary outcome was US turnaround time. Secondary outcomes were percentage of patients requiring invasive interventions to fill the bladder and patients with an US study duration of ≤45 minutes. The percent of patients screened by bladder scan was used as a process measure. Balancing measures used episodes of fluid overload and ED length of stay.

**Results:**

Turnaround time for USs improved from 112.4 to 101.6 minutes. The percentage of patients who had successful USs without invasive bladder filling improved from 32.1% to 42.6%. Bladder volume screening using a bladder scan increased from 40.3% to 82.9%. The successful first-pass US completion rate improved from 77% to 90% consistently.

**Conclusions:**

Through quality improvement methodology, we have identified pelvic US readiness earlier, eliminated some invasive bladder-filling measures, and implemented a rapid fluid protocol. We have sustained these successful results for 2 years. This study can be generalized to any ED with similar patients.

## INTRODUCTION

Adnexal torsion is a surgical emergency and can be seen in female patients presenting to the emergency department (ED) with lower abdominal and pelvic pain. Adnexal torsion accounts for 2.7% of all cases of abdominal pain in children and adolescents.^1^ Although adnexal torsion can occur at any age in the pediatric population, up to 52% occur between 9 and 14 years of age, with a median age of 11 years.^2^ Patients often present with acute onset of unilateral, intermittent, lower abdominal pain. However, the associated signs and symptoms, such as fever, tachycardia, nausea, emesis and the character of pain vary depending on the torsion’s completeness.^3–5^ Due to variable clinical presentations, preoperative imaging confirmation is desired.^6,15^ Radiology performed transabdominal pelvic ultrasound with ovarian Doppler (pelvic US) is the imaging modality of choice for pediatric female patients with concerns of pelvic pathology as it is noninvasive, painless, and radiation-free.^7,8,15^

### Problem Description

A patient’s bladder is used as an acoustic window during pelvic US to evaluate the adnexa and needs to be sufficiently full.^9,10^ The sensation of bladder fullness or arbitrary target volumes were used to identify pelvic US readiness. The patient’s subjective sensation of bladder fullness was often inaccurate,^11–14^ and the target bladder volumes used were too high for children. If the bladder volume was insufficient during pelvic US, patients returned to the ED for indirect bladder filling via intravenous (IV) fluids or direct filling via a Foley catheter. This process can lead to delays in time to diagnosis of adnexal torsion and subsequent delays in surgical intervention, as well as longer ED lengths of stay.^8–10^

There were no established best practices nationwide for pediatric EDs to address this problem. Some pediatric institutions were concurrently working on research studies and workflow improvements to help expedite care for these patients.^11^

In July 2021, we launched a quality improvement (QI) project to expedite care in girls 8–18 of age with concerns for adnexal torsion. Our theory was implementing early objective bladder screening-and rapid bladder-filling strategies would decrease both the turnaround time of pelvic USs and the need for invasive bladder-filling interventions. Our specific aim was to reduce the turnaround time by 25% from 112 to 85 minutes for 2 years among girls 8–18 years of age requiring a pelvic US who present to the ED. Considering the multiple steps involved in the process and based on our past performance, 85 minutes was a pragmatic turnaround time for most patients.

## METHODS

### Setting

Our institution is a 296-bed tertiary care pediatric academic center in Milwaukee, Wisconsin. It is a primary referral site for surrounding urban and rural areas in Wisconsin, Illinois, and Michigan’s upper peninsula. We conducted this project in our level one trauma center, seeing around 71,000 ED visits annually. The ED is staffed by pediatric emergency medicine (PEM) physicians, PEM fellows, advanced practice providers, residents, medical students, physician assistant students, nurses, and ED technicians. On average, 2,272 female patients with abdominal pain present to our ED each year, and approximately 23% undergo a pelvic US to evaluate for adnexal torsion. Of these patients, 2.5% have confirmed adnexal torsion.

### Baseline Process for Pelvic US with Ovarian Doppler

Preintervention, subjective bladder fullness or a predetermined bladder volume threshold of 200–250 mL in children under 13 years and 300–350 mL in children 13 years or older was used to indicate pelvic US readiness. Bladder volume was inconsistently measured using a bladder scan. Patients were transported to the US suite once subjective fullness, or sufficient age-based bladder volume was obtained. If the pelvic structures were well visualized, sonographers completed the pelvic US on the first attempt, and the patient returned to the ED awaiting results and disposition. If the sonographer could not obtain appropriate images due to inadequate bladder volume, the patient was transported back to the ED to await continued bladder filling via IV or Foley catheter. This process continued until appropriate US images were completed. The average turnaround time for pelvic US was 112.4 minutes.

### QI Project

**Fig. 1.**
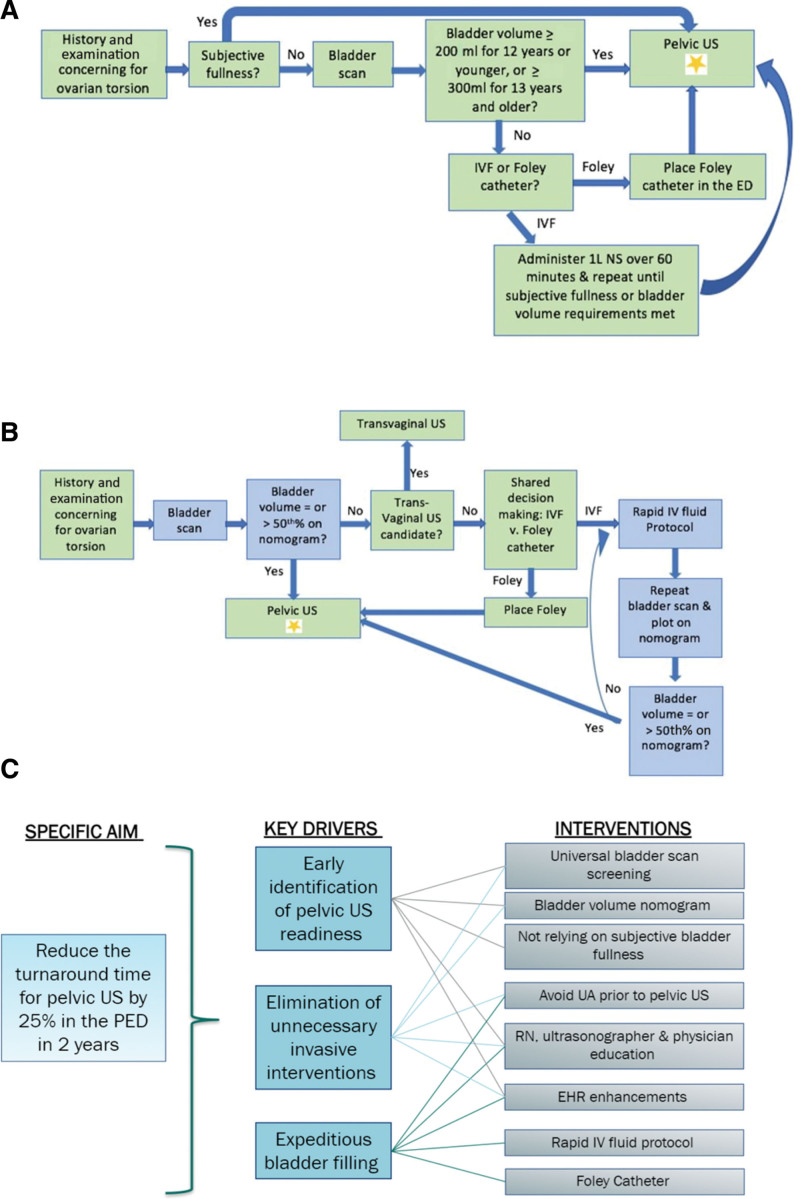
A, Baseline process map. B, Process map during the intervention period. When comparing (A) with (B), the blue boxes in (B) represent the change from the baseline process map. C, Key driver diagram with the specific aim on the left, the three key drivers in the middle column, and the eight main interventions on the right-hand column. IVF, intravenous fluids.

We established a multidisciplinary team, including PEM physicians, pediatric sonographers with a champion sonographer, a nurse and nurse educator, and a pediatric radiologist. A data analyst provided technical support. We reviewed baseline data from January 2020 to June 2021 to create a baseline process map (Fig. 1A). Using feedback from key stakeholders, we developed a revised process map (Fig. 1B) and key driver diagram (Fig. 1C).

Patients included girls 8–18 years of age presenting to the ED with lower abdominal pain who underwent a pelvic US. Patients who were sexually active and agreeable to a transvaginal US were excluded. The key drivers were the early identification of US readiness, eliminating unnecessary invasive interventions, and expeditious bladder filling. Guided by the key drivers, plan-do-study-act ramps were performed to achieve our aim of decreasing the order turnaround time of pelvic USs.

### Implementation

**Fig. 2.**
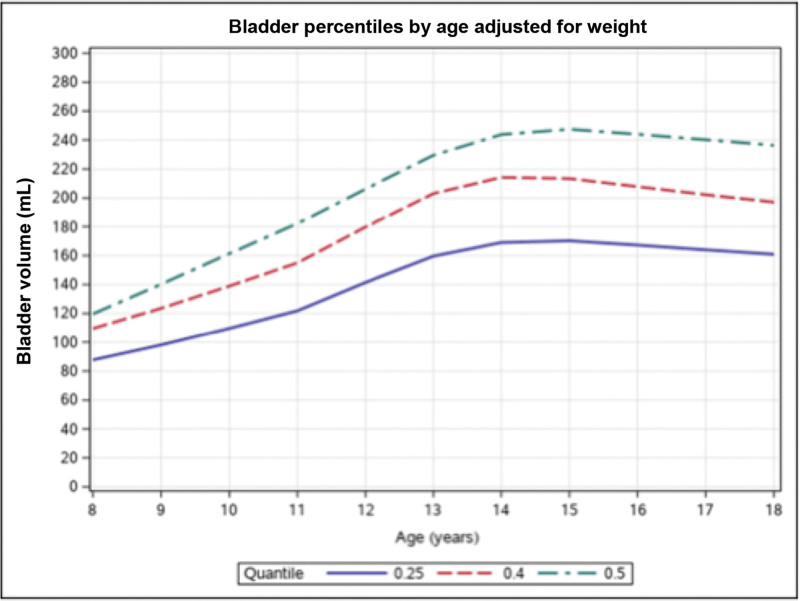
The bladder volume nomogram shows bladder volume percentiles by age, adjusted for weight. The *y* axis represents the bladder volume in milliliters, and the *x* axis represents the age in years.

1. Early identification of US readiness.
  a. Universal bladder screening: Universal bladder scan screening was implemented for all ED patients who needed a pelvic US to confirm adequate bladder fullness. Bladder volume measurement using point-of-care US (POCUS)^12^ was superior to subjective bladder fullness for US readiness. However, we used a bladder scanner (BVI 9400) to measure bladder volume instead of POCUS, as most ED staff were familiar with this machine. All nurses and technicians received training as part of ED orientation and were expected to know how to use the bladder scanner. Nurses quickly realized the benefit of universal bladder screening because it impacted their workload by decreasing the need for IV or Foley catheter placement. When there was a missed opportunity for bladder scan screening, nurses received feedback via email or in person.
  b. Bladder volume nomogram (Fig. 2): An age- and weight-based bladder volume nomogram was developed at Children’s Wisconsin to address the limitations of predetermined bladder volume thresholds. This reference standard was created from a retrospective calculation of 1,030 patient bladder volumes on all successful transabdominal pelvic US with ovarian Doppler studies for patients 8–18 years of age for 2 years. The nomogram has 25th, 40th, and 50th percentile lines. The QI team used the 40th percentile for minimum bladder volume in the initial plan-do-study-act cycles. Upon review of the data and sonographer feedback, some patients had insufficient bladder volume. Most patients had sufficient bladder volumes after changing the threshold to the 50th percentile.
2. Eliminating avoidable invasive interventions: Some patients voided after ED arrival because they were not instructed appropriately. Educational emails were sent to ED staff to avoid ordering a urinalysis before pelvic US and instruct patients to hold urine in anticipation of the pelvic US. Along with universal bladder scan screening, this was expected to decrease the need for invasive interventions to fill the bladder.
3. Expeditious bladder filling: Every patient requiring bladder filling had the option to choose either Foley catheter placement in ED by nursing staff with retrograde filling in the US suite by a sonographer or peripheral IV fluids with secondary bladder filling. Shared decision-making was utilized to choose between a Foley catheter and IV fluids.
  a. Rapid IV fluid protocol: Based on baseline data, patients requesting IV fluids to fill their bladders had the longest order to completion time of pelvic USs. Standard practice in the ED was administering 20 mL/kg (maximum 1-L normal saline) for 60 minutes. Some patients required two or more boluses to fill their bladders sufficiently. A rapid IV fluids protocol was created in collaboration with our nursing team in which 20 mL/kg (up to 1 L normal saline) could be administered for 30 minutes using two IV fluid pumps. Each IV fluid pump was set to deliver 500 mL of normal saline at 999 mL/h into a single IV line. A bladder scan was repeated at the end of the rapid bolus. This process was repeated if the bladder volume was below the 50th percentile.
  b. Electronic health records enhancements: New order panels were created to improve adherence to universal bladder screening and the rapid fluid protocol. These order panels included bladder scan screening, pelvic US orders, rapid fluid orders, and IV pain medications. The rapid fluid orders included the first rapid bolus, a repeat bladder scan order after completing the first bolus, and a second bolus if the bladder volume was insufficient.
  c. Nurse, sonographer, and physician education: Throughout the QI project, ongoing education was maintained via emails and bedside teaching with providers and trainees, huddles, and newsletters with nurses. An email communication was sent monthly to all the trainees working in the ED. Nurses and providers also received on-site feedback and communication about the interventions in the QI project. A lead sonographer facilitated communication with other sonographers.

### Studying the Interventions, Measures, and Outcomes

The primary outcome measure was the pelvic US turnaround time. Pelvic US turnaround time was calculated when the provider placed the pelvic US order to the examination completion time documented by the sonographer. Our secondary outcome measures were (1) the percent of patients who needed invasive interventions such as IV fluids or Foley catheter and (2) the percent of patients with pelvic US examination durations of 45 minutes or less. We used US examination duration, calculated from US start time to completion time as documented by the sonographer, as a surrogate for successful first-pass completion of the pelvic US examination. Forty-five minutes was used as a surrogate based on reviewing prior pelvic USs with our lead sonographer. Our process measure was the percentage of eligible patients screened by the bladder scan. Balancing measures were episodes of fluid overload from the rapid fluid protocol and ED length of stay, defined as patient arrival time to ED discharge time. The outcomes were reported for the entire cohort and after stratification into three patient groups: no Foley or IV fluids, Foley catheter, and IV fluids group.

### Analysis

The patient population was described via descriptive statistics. Statistical process control charts, with control limits set at ±3 SDs, were reviewed weekly to biweekly and used to measure the impact of our interventions. The control limits and centerline were adjusted when eight consecutive points above or below the mean or two of three consecutive points near a control limit were noted. All stratified groups met special cause variation. Single data points outside the control limits were chart reviewed in the electronic medical record to monitor any inconsistencies in typical ED management.

### Ethical Considerations

Our project was considered a QI project and determined to be exempt from informed consent. There were no identified conflicts of interest.

## RESULTS

Our baseline period was from January 2020 to June 2021, and the intervention period was from July 2021 to June 2023. During this project, 1,164 patients between the ages of 8 and 18 underwent pelvic US with a median age of 14. The population included 70.7% White and 19.2% African American patients. Approximately 27% of the population was Hispanic/Latino. From 2020 to 2022, 49 adnexal torsions were diagnosed on US, with 14 cases in 2020 and 2021 and 21 cases in 2022. One case of adnexal torsion was identified through June 2023. Fifteen patients were excluded from the analysis because they received IV fluids and Foley catheters.

**Fig. 3.**
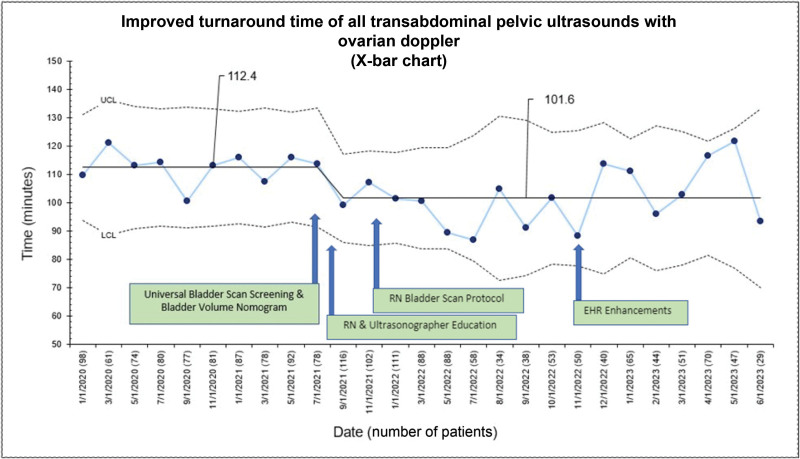
Improved turnaround time of all transabdominal pelvic USs with ovarian Doppler. The *y* axis on this x-bar chart shows the time in minutes. The *x* axis shows the date with the number of patients in parentheses.

**Fig. 4.**
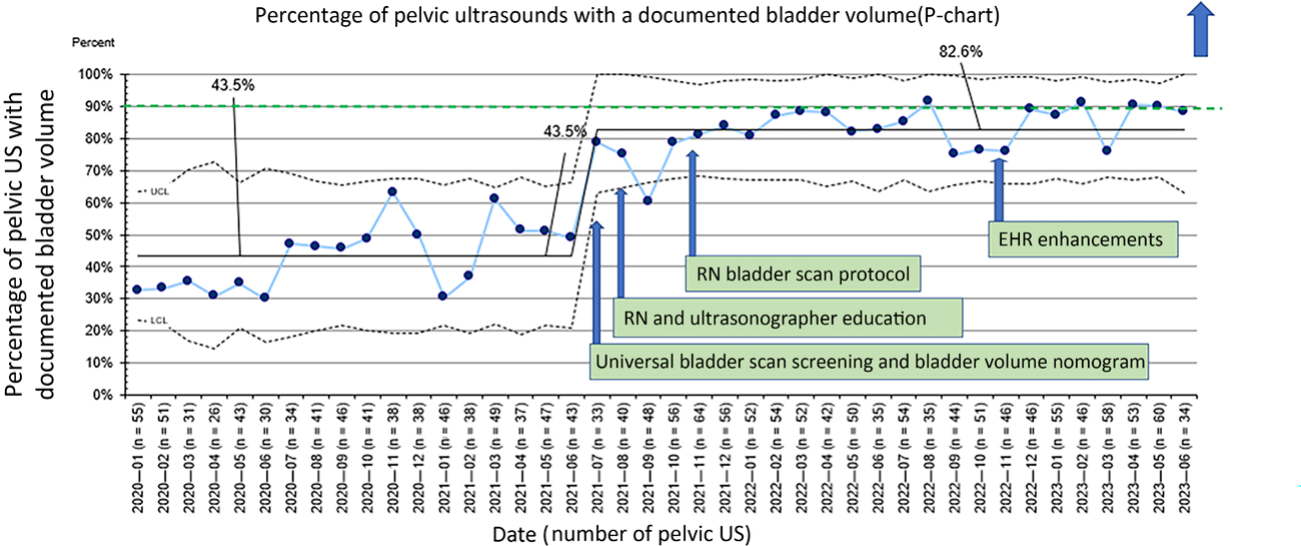
Percentage of pelvic USs with documented bladder volume. This p-chart’s *y* axis shows the percentage of pelvic USs with documented bladder volume. The *x* axis shows the date and the number of pelvic USs in parentheses.

**Fig. 5.**
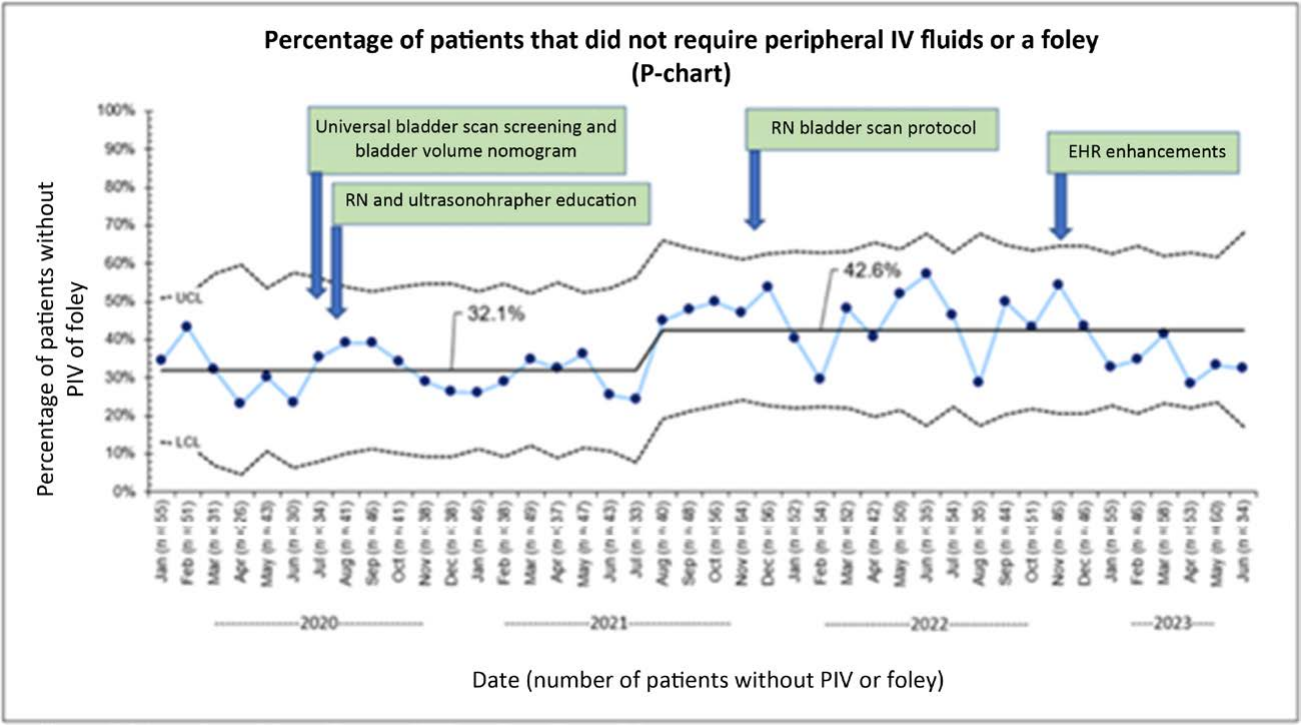
Percentage of patients that did not require peripheral IV fluids or a Foley catheter to fill their bladders. On this p-chart, the *y* axis shows the percentage of patients who do not require a peripheral IV or Foley catheter. The *x* axis is the date with the number of patients in parentheses.

**Fig. 6.**
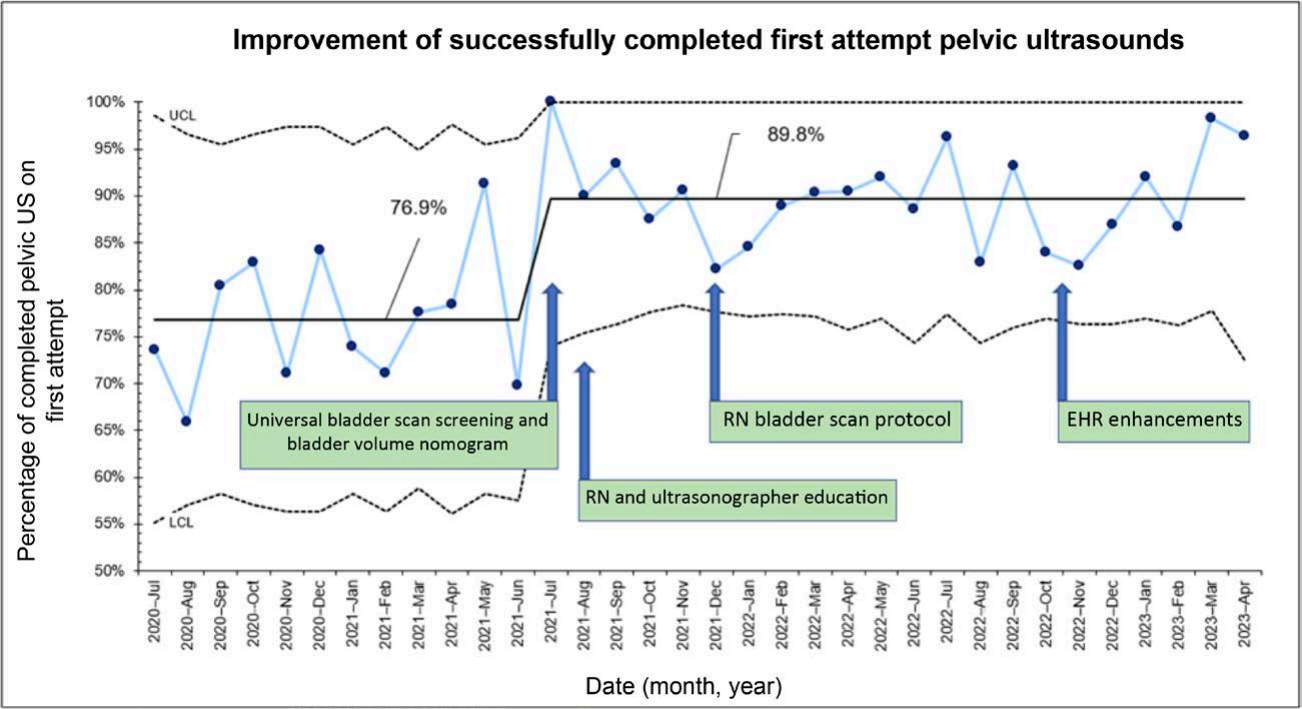
Improvement of completed first-attempt pelvic USs. This p-chart’s *y* axis shows the percentage of completed pelvic USs on the first attempt. The *x* axis shows the date, month, and year.

The pelvic US turnaround time improved from 112.4 to 101.6 minutes and was sustained for nearly 2 years (Fig. 3). Bladder scan screening for pelvic US improved from 40.3% to 82.6% (Fig. 4). When stratified into three subgroups, the turnaround time improved from 74 to 66 minutes for the no interventions group, 96.6 to 82.4 minutes for the Foley catheter group, and 152.9 to 138.3 minutes for the IV fluids group. The percentage of patients who did not require a Foley catheter or peripheral IV fluids increased from 32.1% to 42.6% (Fig. 5). Patients who had pelvic US examination duration of 45 minutes or less improved from 76.8% to 89.5% (Fig. 6).

### Balancing Measure

None of the patients experienced any fluid overload. The ED length of stay for all patients was 334 minutes and remained unchanged. When stratified into three subgroups, the ED length of stay remained unchanged: 280.9 minutes for the no interventions group, 329.4 minutes for the Foley group, and 374.3 minutes for the IV fluids group.

## DISCUSSION

### Summary

Our QI effort led to a modest but sustained improvement in turnaround time for pelvic US and a 10% reduction in the percentage of patients requiring IV fluids or Foley catheters. Before the project, one in four patients had a pelvic US examination duration of 45 minutes or longer, which improved to one in 10. No improvement in ED length of stay was observed during the project period. Universal bladder screening and utilization of age-based bladder volume nomogram to identify pelvic US readiness were the key strength of this QI effort.

### Interpretation

The improved pelvic US turnaround time of 101.6 minutes fell short of our target time of 85 minutes. The 85-minute target was chosen based on the review of our baseline data and the time needed for different steps in the process. The baseline turnaround times were 74 minutes for the no interventions group, 95.9 minutes for the Foley group, and 152.9 minutes for the IV fluids group. By identifying all eligible patients for the no Foley or IV fluids group, we hoped to impact the overall turnaround time. We could identify 10% more eligible patients with universal bladder scan screening and bladder volume nomogram implementation. The improvement in the pelvic US turnaround time of 8 minutes for the no Foley or IV fluids group and 14 minutes for the Foley catheter group was likely attributed to the standardization of different process steps. We anticipated a greater reduction in the turnaround time in the IV fluids group. Our bladder screening rate reached 82.6% and was sustained for more than 18 months. On chart review of patients without documented bladder scan volumes, it was evident the ED team members forgot to document the screening step for most of these patients.

Given these improvements, we anticipated an overall decrease in ED length of stay. The ED length of stay for patients who did not require IV fluids or a Foley catheter was 80 minutes shorter than the average ED length for all patients. Although we increased the number of patients not requiring IV fluids or Foley, it did not impact the overall ED length of stay. Other factors beyond the scope of the project affected the ED length of stay for this patient population, including time to physician in the setting of ED volumes, pain treatment, and specialist consultation.

The bladder volume nomogram was created to improve the successful first-pass completion of pelvic US, but it had not been validated before this project. A pelvic US examination duration of 45 minutes or less was used as a surrogate marker to study this outcome. Implementing the bladder volume nomogram improved this outcome from 77% to 90%. This nomogram was created as a reference standard for pediatric bladder volumes adjusted for age and weight to complete the transabdominal pelvic US successfully. The images reviewed were from prior formal sonographer studies. Ideally, our project would have implemented POCUS to capture patients’ bladder volumes; however, we could not implement POCUS given the limited number of trained staff. In place of POCUS, we used the bladder scanner because it was efficient, and many nurses and ED technicians were trained on it. We could not provide correlation estimates between bladder volumes obtained by bladder scan and pelvic US. However, our project’s improvement in first-pass pelvic US completion offered a pragmatic validation of the nomogram and our method.

During this project, we received positive feedback from the nursing staff and sonographers on multiple new interventions. Through the implementation of universal bladder screening and the nomogram, the nursing workload was reduced because there was a reduction in the number of IVs and Foley catheters placed. Sonographers provided positive feedback as they obtained sufficient images at the 50th% on the nomogram with decreased second studies.

### Limitations

By mirroring the study by Dessie et al,^12^ we defined the pelvic US turnaround time as the time from the ED provider order placement to the completion of pelvic US by sonographers. Numerous workflow processes hinder the time from bladder volume readiness to completion of the image by the sonographer, ultimately delaying turnaround time. Their limitations included concurrent medical resuscitations/level one traumas, US transportation to the US suite, and position in the US queue. We could not track how many patients were sent back to the ED for additional bladder filling so we used pelvic US duration to report a successful first-pass examination completion rate.

### Prior QI Studies

There have been no published QI projects on expediting care for adolescents undergoing an adnexal torsion workup in pediatric EDs. Dr. Park and his QI team^16^ at the University of Washington School of Medicine conducted a similar project simultaneously. Our global aims aligned; however, different interventions resulted in different outcomes. The three main differences in implementation are (1) encouraging per os to aid in bladder filling; (2) bladder volume cutoffs; and (3) rapid IV fluid protocol. With shared interventions, our projects together could provide more successful outcome measures.

## CONCLUSIONS

Through QI methodology, we identified US readiness earlier, eliminated some invasive bladder-filling measures and implemented a rapid fluid protocol. We have sustained these successful results for 2 years. Although this project was performed at a single center, implementation of the nomogram and universal bladder volume screening, alongside the rapid fluid protocol, can be generalized to all EDs that perform pelvic USs for adnexal torsion. In the future, we will consider the use of IV furosemide to help expedite bladder filling^11^ and work closely with urgent care to decrease the number of urinalyses obtained before sending patients in hopes these patients will have full bladders upon ED arrival.
